# Biomarker-Driven Strategies for Stromal Reprogramming in Pancreatic Ductal Adenocarcinoma

**DOI:** 10.3390/cells15181637

**Published:** 2026-09-10

**Authors:** Maria De Luca, Francesco Balestra, Giorgia Panzetta, Gianluigi Giannelli, Maria Principia Scavo

**Affiliations:** 1 Laboratory of Molecular Medicine, National Institute of Gastroenterology IRCCS de Bellis, Via Turi 27, Castellana Grotte, 70013 Bari, Italy; maria.deluca@irccsdebellis.it (M.D.L.); francesco.balestra@irccsdebellis.it (F.B.); giorgia.panzetta@irccsdebellis.it (G.P.); 2 Scientific Direction, National Institute of Gastroenterology IRCCS de Bellis, Via Turi 27, Castellana Grotte, 70013 Bari, Italy; gianluigi.giannelli@irccsdebellis.it

**Keywords:** pancreatic ductal adenocarcinoma, stromal reprogramming, tumor microenvironment, biomarkers, cancer-associated fibroblasts, extracellular matrix

## Abstract

Pancreatic ductal adenocarcinoma (PDAC) remains highly lethal, partly because its dense, heterogeneous stroma restricts drug delivery, promotes immune exclusion, and supports therapeutic resistance. Stromal targeting has, therefore, evolved from broad depletion towards biomarker-guided reprogramming and normalization strategies that aim to restore vascular function, improve intratumoral drug penetration, and enhance antitumor immunity. This review summarizes evidence on stromal biomarkers with prognostic or predictive value, including tumor–stroma ratio, fibrosis burden, cancer-associated fibroblast states, extracellular matrix stiffness, vascular accessibility, and spatial immune features. It also examines emerging therapeutic approaches to reprogram the PDAC stroma, such as repurposed drugs, immune–stromal modulators, metabolic interventions, and advanced delivery systems. Emphasis is placed on clinically or translationally supported strategies, including CXCR4, CD40, CD73, vitamin D receptor agonism, matrix remodeling, and biomarker-driven stratification. Overall, PDAC stroma is a dynamic therapeutic target, requiring composite biomarkers and rational combinations with chemotherapy, immunotherapy, and delivery-enhancing strategies.

## 1. Introduction

Pancreatic ductal adenocarcinoma (PDAC) is among the deadliest solid tumors, with an overall 5-year survival rate of approximately 12–13% and of only ~3–4% in metastatic disease [1]. Despite three decades of therapeutic escalation, from gemcitabine to FOLFIRINOX and gemcitabine/nab-paclitaxel, survival gains have remained limited [2,3].

This refractoriness is driven not only by malignant epithelial cells harboring key oncogenic alterations (e.g., KRAS mutations, TP53 loss, SMAD4 inactivation) but also by the surrounding desmoplastic stroma. PDAC stroma constitutes a substantial portion of the tumor mass and comprises extracellular matrix components (e.g., type I/III collagen, hyaluronan) and diverse cellular populations, including cancer-associated fibroblasts, tumor-associated macrophages, myeloid-derived suppressor cells, regulatory T cells, aberrant vasculature, and neural elements [4].

CAFs are a key stromal population comprising both heterogeneous subsets with distinct phenotypic and functional properties, including myofibroblastic (myCAFs), inflammatory (iCAFs), antigen-presenting (apCAFs), LRRC15-positive, inflammatory-signature, and tumor-restraining fibroblasts [5] that together establish multiple layers of therapeutic resistance. CAF subsets can adapt in response to therapeutic pressure by remodeling the composition of ECM, increasing tissue stiffness, promoting epithelial to mesenchymal transition (EMT), and supporting the formation of metastatic niches [6].

At the physical level, an excess in ECM deposition and a hyaluronan-mediated increase in interstitial fluid pressure compress blood vessels, thereby impairing perfusion and limiting drug delivery [7]. Chronic hypoxia promotes hypoxia-inducible factor (HIF)-1α-dependent glycolysis, autophagy, and adaptation to nutrient deprivation. Concurrently, TGF-β- and CXCL12-enriched stromal gradients contribute to the exclusion of CD8^+^ T cells from tumor nests, while additional immunosuppressive mechanisms such as regulatory T cell activity, IDO1 signaling, and the intrinsically low immunogenicity of PDAC further limit the efficacy of immune checkpoint blockade in unselected patients [8]. Single-cell and spatial profiling show that PDAC stroma is a plastic, heterogeneous ecosystem, explaining paradoxical effects of broad depletion or single-pathway inhibition, such as TGF-β. Stromal resistance is biomarker-defined and spatially organized, driving normalization, redirection, and reprogramming rather than indiscriminate removal [9]. In this context, stromal reprogramming has emerged as a central therapeutic concept in PDAC. However, the limited clinical success of broad stromal depletion has highlighted the biological complexity and context-dependent functions of the PDAC stroma, in which individual stromal components may exert both tumor-promoting and tumor-restraining effects. Consequently, selective stromal reprogramming or normalization may provide a more rational therapeutic strategy than indiscriminate stromal removal. A central objective of this review is, therefore, to critically evaluate which stromal features can serve as clinically actionable biomarkers for patient selection and to distinguish strategies supported by clinical evidence from those that remain predominantly translational or preclinical.

Current strategies include repurposed drugs, semaphorin-targeted approaches, advanced nanotechnology-based delivery systems, PROTAC-enabled platforms, CAR-T and gene-based therapies, biomarker-guided cytotoxic treatment, multimodal immune–stromal combinations, and metabolic interventions aimed at restoring immune activation. Ongoing clinical trials continue to assess these approaches across stromal and molecular PDAC subgroups. Together, these advances reflect a shift towards integrated stromal reprogramming and delivery-oriented therapy in PDAC. This review synthesizes evidence on stromal biomarkers, reprogramming strategies, and delivery-enabled approaches designed to overcome the physical, metabolic, and immunological barriers of the PDAC microenvironment.

## 2. Materials and Methods

### 2.1. Review Design

This article is a narrative review informed by a structured literature search. The aim is to critically synthesize the available evidence on stromal biology, stromal biomarkers, and stromal-directed therapeutic strategies in PDAC, with particular emphasis on clinically and translationally relevant findings. The search and selection process were structured to ensure a broad and transparent identification of the relevant literature.

### 2.2. Information Sources and Structured Search Strategy

A structured literature search was performed in PubMed, Embase, and ClinicalTrials.gov to identify publications and registered clinical studies relevant to PDAC stromal biology and therapeutic targeting. The primary search covered studies published between April 2020 and April 2026. Seminal studies published before 2020 were also considered when they provided essential biological, methodological, or historical context for the topics discussed in this review. The search strategy combined terms related to PDAC, the tumor microenvironment, stromal biology, biomarkers, and therapeutic interventions. The principal search concepts included the following terms related to PDAC, stromal biology, and therapeutic intervention: (“pancreatic ductal adenocarcinoma” OR “pancreatic cancer” OR “PDAC”) AND (“stroma” OR “tumour microenvironment” OR “cancer-associated fibroblast” OR “CAF” OR “fibrosis” OR “desmoplasia” OR “extracellular matrix”) AND (therapy* OR treatment* OR target* OR reprogram* OR remodel* OR biomarker* OR “drug delivery” OR immunotherapy* OR nanotechnology* OR PROTAC). Additional focused searches were conducted to identify the literature concerning specific stromal features, molecular pathways, therapeutic agents, and delivery platforms. These searches included the following terms and topics: tumor–stroma ratio, stromal stiffness, ITGB5, hENT1, SMAD4, CXCR4, CD40, CD73, semaphorins, all-trans retinoic acid, paricalcitol, losartan, metformin, statins, chloroquine, ascorbic acid, ketogenic diet, nanoparticles, thermosensitive liposomes, focused ultrasound, CAR-T cells, TCR-T cells, and KRAS-directed T cell therapy. The search was complemented by screening the reference lists of relevant publications and reviews to identify additional studies that were considered important for the biological or translational context of the review. Reviews were used primarily to identify relevant primary studies and to provide background information; they were not considered equivalent to primary evidence in the narrative synthesis.

### 2.3. Eligibility Criteria

Studies were considered relevant when they addressed PDAC cohorts, PDAC models, or PDACrelated translational systems and investigated one or more of the following areas: stromal biology or tumor microenvironment features; cancer-associated fibroblast populations or states; fibrosis, desmoplasia, extracellular matrix composition, or matrix stiffness; vascular accessibility, tissue perfusion, or drug delivery; immune–stromal interactions and immune exclusion; metabolic or hypoxia-related features of the tumor microenvironment; biomarkers associated with prognosis, treatment response, therapeutic vulnerability, or patient stratification; therapeutic strategies targeting stromal, immune, metabolic, vascular, or extracellular matrix components; stromal reprogramming, stromal normalization, or delivery-enhancing approaches; and nanotechnology-based delivery systems, PROTAC-based platforms, cellular therapies, or gene-based approaches with potential relevance to the PDAC microenvironment. Priority was given to randomized clinical trials, prospective studies, phase I–III clinical trials, multicenter investigations, and well-annotated retrospective studies, particularly those providing clinically relevant outcomes or biomarker information. Observational studies and smaller early-phase studies were included when they provided relevant clinical, biological, or translational information. Selected preclinical studies were also considered when they offered important mechanistic insights, identified potentially actionable stromal pathways, or described innovative therapeutic or drug delivery strategies that had not yet been adequately evaluated in clinical studies.

### 2.4. Literature Selection and Thematic Organization

The search retrieved 7321 records across the selected information sources. After removal of duplicate records and screening for relevance, 331 publications were identified as potentially relevant to the subject of the review. These publications constituted the evidence pool used to inform the subsequent thematic analysis. Because the present work was designed as a narrative review rather than as a systematic evidence map, the 331 identified publications were not treated as a uniformly included dataset, and no attempt was made to provide an exhaustive study-by-study accounting of all records in the main manuscript. Instead, the available literature was organized thematically according to the principal biological and therapeutic domains relevant to PDAC stromal reprogramming.

The final narrative synthesis focused on representative studies that best illustrated the physical, immune, neural, and metabolic mechanisms underlying stromal resistance; clinically relevant stromal biomarkers and biomarker-guided stratification; pharmacological strategies aimed at stromal reprogramming or normalization; metabolic, autophagy-related, and oxidative stress interventions; biotechnological and nanotechnology-based strategies for overcoming stromal and immune access barriers; cellular, gene-based, and delivery-enhancing approaches; and the current clinical translation of stromal-directed interventions. The selection of studies for detailed discussion was based on scientific relevance, methodological quality, clinical or translational significance, novelty, and their ability to contribute to the conceptual framework of the review.

Beyond study selection, the available evidence was critically compared according to the level of evidence, stage of clinical development, degree of biomarker validation, and major limitations affecting translational and clinical applicability.

## 3. Results

### 3.1. Mechanisms of Stromal Resistance: Physical, Immune, Neural, and Metabolic Barriers

**Figure 1:**
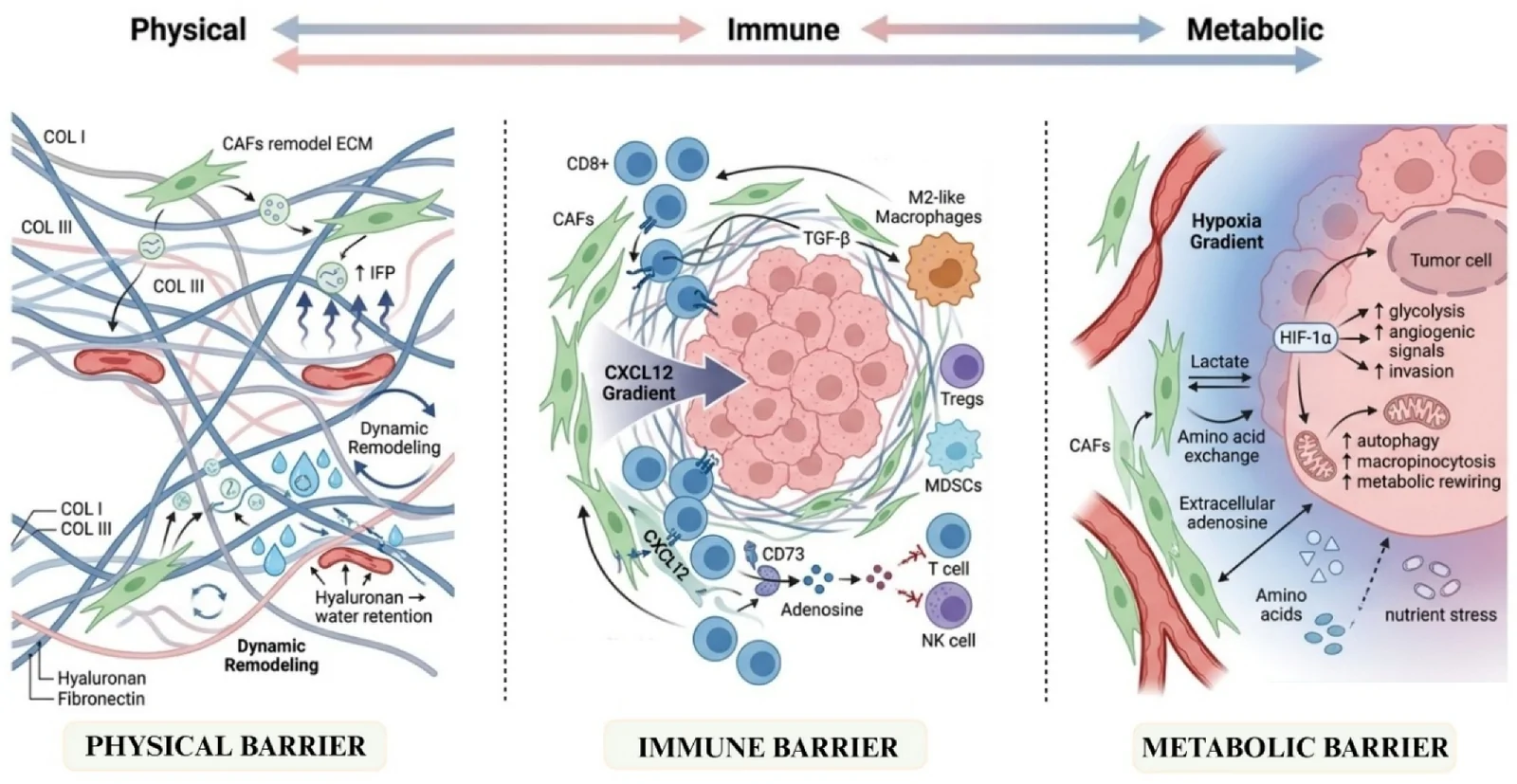
Physical, immune, and metabolic stromal barriers in PDAC. Schematic overview of the interconnected extracellular matrix remodeling, immune exclusion/suppression, and hypoxia-driven metabolic adaptations that limit drug delivery and therapeutic response in the PDAC microenvironment.

The therapeutic refractoriness of PDAC is strongly influenced by the ability of the stromal compartment to generate multiple, interconnected resistance barriers (Figure 1). The dense ECM, enriched in fibrillar collagens, particularly types I and III, hyaluronan, and fibronectin, forms a mechanically rigid scaffold that increases tissue stiffness, elevates interstitial fluid pressure (IFP), and contributes to impaired drug delivery [10]. The accumulation of hyaluronan exacerbates mechanical stress, promoting interstitial water retention that leads to vascular compression and severely compromises both microvascular perfusion and the convective delivery of cytotoxic therapies, despite adequate circulating levels [11]. Crucially, this barrier is a dynamic scaffold remodeled by plastic CAFs, which adapt to therapy. Depending on activation state and spatial distribution, CAF subsets may promote or restrain progression by depositing and reorganizing ECM, modulating cross-linking, and regulating vascular access. These mechanisms support stromal normalization rather than indiscriminate depletion [12].

A second major layer of stromal resistance involves immune exclusion and suppression. A key stromal resistance mechanism is immune exclusion, with CD8^+^ T cells confined to the stromal periphery by CAF-driven chemokines, immunosuppressive myeloid cells, Tregs, and polarized macrophages [13]. Tumor-associated macrophages (TAMs), which frequently exhibit an M2-like phenotype, reinforce an immunosuppressive microenvironment by promoting tumor progression and limiting effective antitumor immune responses. Evidence from non-PDAC tumor models provides additional insight into their functional plasticity. For example, studies in osteosarcoma suggest that macrophage repolarization toward an M1-like state may represent a potential therapeutic strategy [14]. Similarly, observations in hepatocellular carcinoma illustrate how TAMs, CAFs, fibrosis, and immunosuppressive mediators may act cooperatively to promote treatment resistance [15]. These findings are included as supportive extrapolations and should not be interpreted as direct evidence of therapeutic activity in PDAC. In PDAC, the CXCL12/CXCR4 axis restricts T cell infiltration and may impair responses to immunotherapy, whereas TGF-β promotes fibroblast activation, extracellular matrix deposition, epithelial–mesenchymal transition, and T cell dysfunction, thereby linking desmoplasia with immune suppression [16,17]. Additional metabolic–immunological mechanisms may contribute to immune suppression in PDAC liver metastases. In particular, CD73-mediated adenosine production can inhibit T and NK cell activity while promoting regulatory and immunosuppressive myeloid programs, thereby potentially contributing to resistance to immune checkpoint blockade [18].

Metabolic stress represents a third, closely integrated stromal barrier. The combination of compressed vasculature, poor perfusion, and high cellular demand generates chronic hypoxia and nutrient deprivation within the PDAC microenvironment [10]. Hypoxia stabilizes HIF-1α and promotes transcriptional programs that enhance glycolysis, angiogenic signaling, epithelial plasticity, invasion, and resistance to therapy. At the same time, nutrient limitation and oxidative stress favor adaptive survival mechanisms such as autophagy, macropinocytosis, mitochondrial rewiring, and metabolic cooperation between cancer cells and stromal compartments [19]. CAFs and immune cells shape metabolic constraints (lactate, redox, amino acids, adenosine), supporting interventions such as autophagy inhibition, oxidative stress modulation, nutrient targeting, and high-dose ascorbate [20]. However, metabolic targeting requires biomarker integration (hypoxia, stroma, immunity, redox).

Finally, stromal resistance in PDAC also involves neural and axon guidance pathways, including semaphorin-mediated signaling, key context-dependent regulators of the PDAC microenvironment [21]. In this type of cancer, semaphorin signaling may influence multiple stromal and non-stromal compartments, including CAFs, nerves, macrophages, endothelial cells, and tumor epithelial cells. Rather than acting as uniformly tumor-suppressive or tumor-promoting mediators, the biological consequences of semaphorin signaling appear to depend on ligand–receptor pairing, cellular source, receptor distribution, tumor subtype, and spatial localization within the tumor microenvironment [22]. In PDAC, tumor-derived SEMA3A promotes neuronal recruitment and is associated with enhanced tumor growth and dissemination [23]. Particularly, SEMA3A promotes aggressiveness, chemoresistance, and metastatic traits in pancreatic cancer via the FAK signaling pathway, and fosters an immunosuppressive microenvironment, suggesting that inhibiting SEMA3A or tumor-associated macrophages can enhance chemotherapy efficacy [21]. Indeed, pancreatic cancer cell-derived SEMA3A has been shown to promote neuronal recruitment and accelerate tumor growth and dissemination, supporting a role for SEMA3A in the neurotropic and invasive biology of PDAC [23,24]. In line with this interpretation, SEMA3A has also been implicated in the aggressive phenotype of basal-like PDAC [21]. Semaphorin-mediated crosstalk shapes neuro-immune organization in PDAC, with M2-like macrophages enriched near SEMA3D-expressing nerves, suggesting tumor hijacking of neural signaling to promote invasion and immune suppression. The semaphorin axis may define distinct stromal, neural, and immune states, requiring validation through spatial transcriptomics (e.g., GeoMx, Xenium) to map ligand–receptor interactions within tissue architecture [25]. Such precision is essential for characterizing the biological heterogeneity of semaphorin axes and confirming their clinical relevance. However, prospective clinical validation is still required before semaphorin-based biomarkers, including baseline PlexinA4 expression, can be used to guide patient stratification or predict therapeutic benefit [21]. Taken together, these observations highlight that stromal heterogeneity and CAF plasticity are major determinants of the variable response to stromal-directed therapies. The coexistence of tumor-promoting and tumor-restraining stromal states may partly explain why indiscriminate stromal depletion or inhibition of a single pathway has produced limited or context-dependent therapeutic benefit. Accordingly, effective stromal targeting should aim to selectively reprogram pathogenic stromal states while preserving potentially protective functions. This reinforces the need for context-dependent, biomarker-guided approaches capable of identifying the stromal features that are most relevant to therapeutic resistance in individual patients.

### 3.2. Therapeutic Strategies

**Figure 2:**
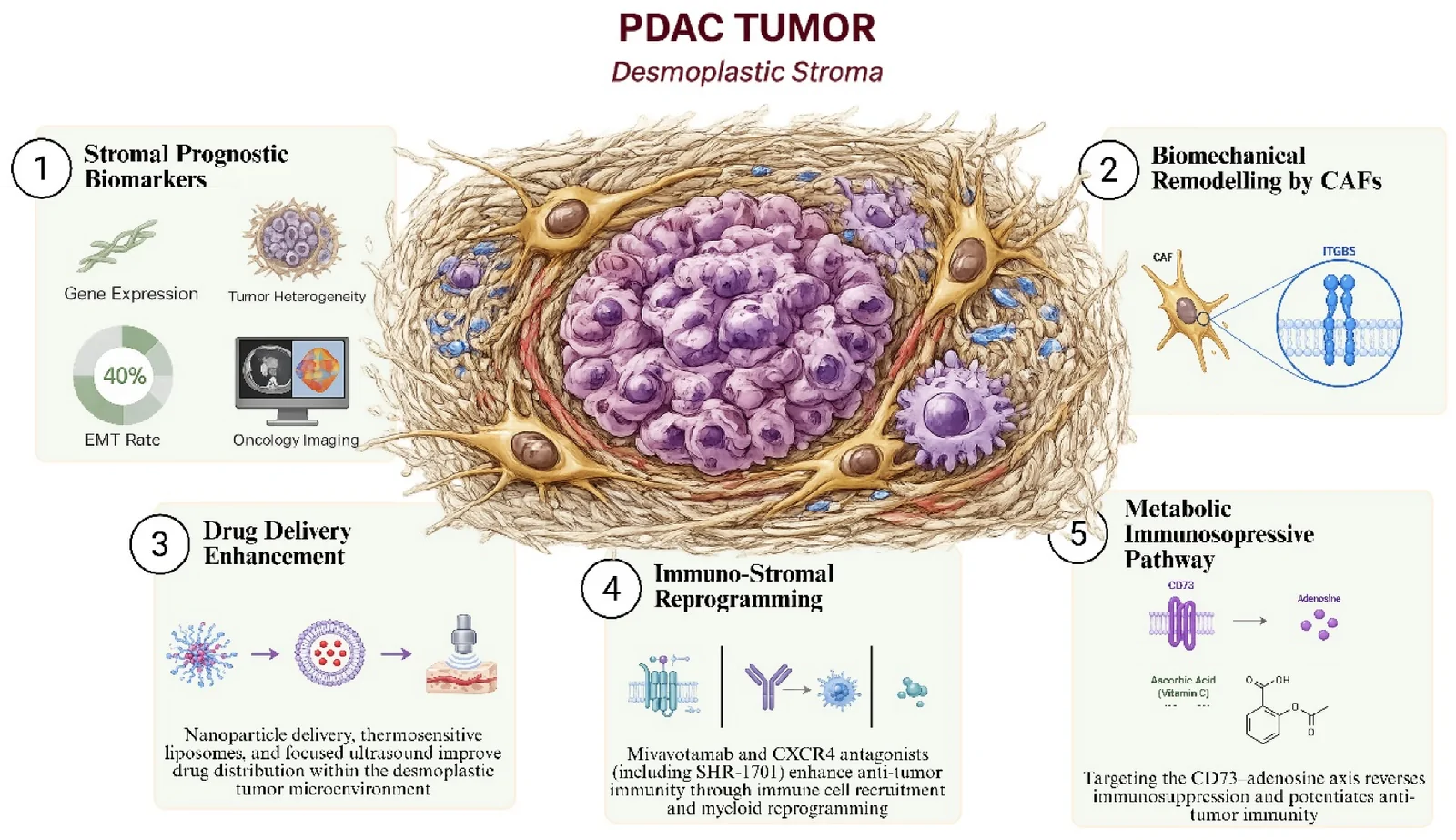
Overview of stromal therapeutic strategies in PDAC. Schematic representation of the desmoplastic PDAC stroma and major biomarker-guided, biomechanical, drug delivery, immune–stromal, and metabolic approaches aimed at reprogramming the tumor microenvironment and improving therapeutic response.

Increasing evidence indicates that stromal density and fibrosis are prognostic in PDAC, impairing drug delivery and survival; collagen deposition, especially at ≥40%, stratifies patients [26]. Furthermore, the spatial organization of collagen fibers, particularly their alignment at the tumor–stroma interface, has emerged as an additional determinant of poor clinical outcome, highlighting the importance of both the quantity and architecture of the ECM in PDAC progression [27]. Several stromal-directed strategies aim to improve drug penetration; in the PanDox phase I trial, stromal-directed strategies enhance drug penetration. The PanDox trial uses ultrasound-triggered thermosensitive liposomal doxorubicin for unresectable PDAC, while CT-based radiomics enables non-invasive stromal burden and prognostic stratification [28]. In parallel, CT-based radiomics has been proposed as a non-invasive method to stratify stromal burden and prognosis before treatment [29]. Other approaches include all-trans retinoic acid combined with gemcitabine and nab-paclitaxel for locally advanced diseases [30]. Immune–stromal crosstalk represents a key therapeutic axis, as illustrated by CXCR4 antagonists plus PD-1 blockade, CD40 agonists (e.g., mitazalimab with mFOLFIRINOX), and SHR-1701 with gemcitabine/nab-paclitaxel, highlighting roles for macrophage compensation, fibrosis-related signatures, and biomarkers (pSMAD2/3 and PD-L1) [31]. Metabolic and adenosine-related pathways also contribute to stromal immunosuppression, as shown by CD73 inhibition with quemliclustat combined with chemotherapy and PD-1 blockade [32]. In a similar translational framework, high-dose ascorbic acid combined with chemotherapy was tolerable in metastatic PDAC, although it did not clearly improve outcomes over historical controls [33]. The recent phase III RASolute 302 trial (NCT06625320) supports RAS-targeted therapy in metastatic PDAC, which should be integrated with biomarker-guided stromal, immune, and drug delivery strategies to overcome microenvironment-driven resistance [34]. These therapeutic interventions in PDAC are summarized in the following Figure 2.

These studies indicate that stromal interventions in PDAC are shifting from broad depletion to selective reprogramming, requiring biomarkers to define prognostic, targetable states and predict responses to microenvironment-directed therapies. Most importantly, the therapeutic strategies discussed in this review differ substantially in their level of evidence and stage of clinical development, ranging from approaches evaluated in prospective clinical trials to early translational and predominantly preclinical concepts. In order to avoid mixing biological promises with established clinical benefit, these strategies are critically distinguished throughout the review according to their degree of clinical validation, supporting biomarker evidence, and translational maturity. Particular emphasis is placed on separating clinically evaluated interventions from emerging approaches whose therapeutic relevance is still to be confirmed in prospective studies.

#### 3.2.1. Biomarker-Guided Stromal Stratification

Biomarker-guided stromal stratification identifies PDAC features predicting prognosis, vulnerability, and response, including fibrosis, TSR, CAF states, ECM stiffness, vascular access, immune exclusion, and immunosuppressive pathways [35]. Emerging liquid biopsy approaches further suggest that stromal components such as circulating CAFs and plasma hyaluronan may provide clinically accessible biomarkers of stromal burden and prognosis [36].

TSR is a practical and clinically translatable metric, with low TSR associated with lymph node metastasis, poorer disease-free survival, and distinct mutational profiles across surgical specimens, EUS-FNA biopsies, and pre-treatment samples [37]. Importantly, TSR serves as a prognostic marker and surrogate of stromal organization, reflecting epithelial cellularity, desmoplasia, and immune access. Stromal composition links to molecular programs, with CAF-driven ITGB5-mediated matrix stiffness highlighting integrin-dependent tension as a target for stromal normalization. Transcriptomic and multimodal analyses identified several CAF-associated hub genes; among them, ITGB5 emerged as a key regulator of stromal biomechanics. Its targeting reduced tissue stiffness in preclinical models and may improve chemotherapy response, although clinical benefit remains unproven [38]. In a prospective single-center trial, stromal stiffness correlated with CAF activation and worse outcomes, showing that PDAC stroma drives progression through ECM remodeling, fibrosis, and immune exclusion. The intratumoral microbiome further shapes CAF phenotypes, collagen deposition, and T cell exclusion [39]. This distinction is clinically relevant, as PDAC stroma can promote or restrain tumor progression depending on CAF subtype, matrix architecture, vascular function, and immune context, while matrix remodeling can influence both tissue stiffness and tumor perfusion [40]. No single biomarker is likely to capture this complexity; multidimensional approaches integrating stromal, molecular, spatial, and proteomic features may, therefore, provide a more robust framework for patient stratification and therapeutic decision-making [41,42]. Table 1 provides a schematic overview of representative biomarkers and their biological and clinical implications.

##### Biomarker Validation and Clinical Implementation

The transition from exploratory stromal biomarkers to clinically actionable stratification tools requires rigorous analytical and clinical validation. Candidate biomarkers should demonstrate reproducibility across observers, laboratories, and pre-analytical conditions before standardized cut-offs are established. This is particularly important for histological and imaging-derived features such as tumor–stroma ratio, fibrosis, CAF abundance, and immune cell density, which can vary with sampling, segmentation, and scoring methods. Cut-offs should, therefore, be prespecified and independently validated, as retrospectively optimized thresholds may be overfitted and poorly transferable across institutions, disease stages, and specimen types [43]. A further challenge is that the PDAC microenvironment is intrinsically spatially and temporally heterogeneous. Spatial transcriptomic analyses of matched primary and metastatic PDAC have demonstrated the coexistence of distinct fibrotic, metabolic, and immunosuppressive ecotypes, with differences not only within individual lesions but also between primary tumors and metastatic sites [44]. Additional spatial studies have shown marked regional heterogeneity in tumor–cell states, immune organization, and treatment-associated microenvironmental remodeling [45,46]. A stromal measurement from a single tumor region may not represent the entire lesion or disease at other sites. This limitation also applies over time, as treatment can alter CAF phenotypes, immune cell distribution, matrix architecture, and tumor programs. Biomarkers should, therefore, ideally be assessed near the treatment decision point, with longitudinal tissue, imaging, or circulating markers providing complementary information when feasible. Biopsy representativeness is especially relevant in PDAC, where treatment decisions often rely on limited EUS-guided samples. Although fine-needle biopsies can reproduce several molecular targets detected in surgical specimens, concordance is marker-dependent and sampling error remains a limitation [47]. Encouragingly, recent tumor–stroma ratio studies have demonstrated reproducibility of clinically relevant associations across surgical specimens, validation cohorts, EUS-FNA samples, and pre-treatment biopsies, indicating that carefully selected stromal measurements can potentially be transferred to routine biopsy material [37]. Architecture-dependent biomarkers such as TSR, collagen organization, spatial immune exclusion, and CAF distribution are particularly sensitive to sample size and location. Multi-region sampling, standardized tissue requirements, digital pathology, and whole-lesion imaging may help reduce this bias. It is also essential to distinguish prognostic from predictive biomarkers. Most stromal markers in PDAC currently identify aggressive disease or poor survival but do not necessarily predict benefit from stromal-targeted therapy. Predictive utility requires prospective evidence that treatment effects differ according to biomarker status, ideally in randomized trials with formal biomarker–treatment interaction analyses [43]. This distinction is particularly relevant for fibrosis, TSR, CAF signatures, ECM stiffness, immune exclusion, and CXCR4-, TGF-β-, or adenosine-related pathways, whose predictive value for stromal-targeted therapies remains uncertain. Prospective validation should, therefore, test candidate classifiers in independent, preferably multicenter cohorts using prespecified assays, cut-offs, endpoints, and analytical plans. Biomarker-driven trials should also incorporate longitudinal pharmacodynamic measures to confirm that treatment modifies the stromal feature identified by the biomarker. Clinical implementation also requires assays that are reproducible, affordable, rapid, and feasible with routinely available tissue. Although spatial transcriptomics and multi-omics are powerful discovery tools, their complexity and cost may limit routine use. A practical strategy may, therefore, translate high-dimensional signatures into simpler digital pathology, immunohistochemical, targeted molecular, or imaging-based assays. Given the biological complexity of PDAC, composite biomarkers integrating stromal abundance, CAF states, matrix properties, perfusion, and immune accessibility are likely to be more informative than individual markers. Ultimately, clinically useful classifiers should identify actionable stromal states and guide treatment selection rather than simply describe stromal abundance [44,46].

### 3.3. Repurposed Pharmacological Strategies

Among the studies retrieved through the structured search, 19 investigations were selected as representative examples of repurposed strategies aimed at overcoming stromal and efflux-mediated therapeutic resistance in PDAC. Because the therapeutic rationale differs across studies, their specific mechanisms of action are not discussed in a single undifferentiated list but rather are briefly organized and schematized in the two paragraphs that follow.

#### 3.3.1. Clinically Tested Stromal-Reprogramming

All-trans retinoic acid (ATRA) combined with gemcitabine and nab-paclitaxel, as evaluated in the STARPAC trial, demonstrated feasibility and encouraging activity compared with standard therapy, supporting further randomized evaluation in STARPAC-2/PRIMUS-005 [30]. In another study, paricalcitol (vitamin D receptor agonist) with pembrolizumab (phase II) modulated the tumor microenvironment (increased CD8^+^ T cells, decreased regulatory T cells) despite lacking significant progression-free survival (PFS) benefit [48]. Preliminary clinical and translational evidence suggests biomarker-stratified benefits (fibrosis ≥ 40%, hENT1-high), with HR for PFS/OS < 0.75, supporting stromal normalization [49]. FOLFIRINOX plus losartan reduced collagen, stiffness, and improved gemcitabine penetration [50,51,52]. LOX-mediated collagen stabilization increases tissue stiffness and interstitial fluid pressure, which in turn impairs vascular perfusion and limits the delivery of chemotherapy. While early clinical efforts with the LOXL2-specific antibody simtuzumab failed to improve survival in metastatic PDAC [53], newer approaches targeting the entire LOX family have shown promise [40]. This mechanical normalization represents a more refined potential strategy compared to total stromal depletion, as it aims to restore the physiological balance of the matrix to facilitate drug access without losing the tumor-restraining properties of the stroma.

#### 3.3.2. Metabolic/Autophagy/Oxidative Stress Repurposing

Although primarily described as a stromal-targeting strategy, ATRA-mediated stromal modulation may also intersect with treatment-induced cellular stress responses, as changes in CAF activation were accompanied by broader proteomic alterations, including the downregulation of RNA-processing proteins following neoadjuvant chemotherapy [54]. Similarly, high-dose intravenous ascorbic acid (56.25 g/m^2^) with nab-paclitaxel, 5-fluorouracil/leucovorin, and gemcitabine (phase Ib) [34] proved tolerable without reaching a maximum tolerated dose. The regimen achieved a PFS of 7.1 months and an OS of 14.2 months, comparing favorably with historical controls (6–10 months), potentially through glutathione depletion in KRAS-mutant cells and a reduction in hepatic steatosis on textural imaging [33]. Beyond these studies, several additional repurposed strategies have been explored in PDAC. Metformin use was associated with significantly improved overall survival in patients undergoing upfront surgery (median OS 29 vs. 14 months; adjusted HR ≈ 0.56), but not in those receiving neoadjuvant chemoradiotherapy. Transcriptomic analysis of the tumor microenvironment revealed reduced immunosuppressive M2 macrophages and increased immuno-activating dendritic cells in metformin users, suggesting enhanced antitumor immunity as a potential mechanism. These findings support the hypothesis that metformin may confer prognostic benefits in selected PDAC subgroups, particularly when the immune microenvironment has not been profoundly altered by neoadjuvant therapies [53,55]. Hydroxychloroquine combined with gemcitabine and nab-paclitaxel has been associated with a median progression-free survival (PFS) of approximately 4 months in patients with advanced solid tumors, whereas the median PFS of 5.5 months is more appropriately attributed to first-line gemcitabine plus nab-paclitaxel, as reported in advanced pancreatic cancer [56,57]. A randomized phase II trial of a medically supervised ketogenic diet (MSKD) combined with gemcitabine, nab-paclitaxel, and cisplatin in advanced PDAC induced nutritional ketosis in 94% of patients (median β-hydroxybutyrate 0.5–3.0 mM for 39.4% of days) and was associated with numerically longer median PFS (8.5 months; HR 0.53, 95% CI 0.21–1.37, one-sided *p* = 0.096) and OS (13.7 months; HR 0.58, 95% CI 0.25–1.37, *p* = 0.107), together with beneficial microbiome enrichment (*p* < 0.05) and no incremental toxicity [58]. A leading pharmacological strategy for the mechanical normalization of the PDAC extracellular matrix involves the inhibition of the lysyl oxidase family, which is responsible for the copper-dependent cross-linking of collagen and elastin fibers [59].

#### 3.3.3. Observational or Early/Preclinical Repositioning Signals

Glucocorticoids have also been investigated as repurposed agents in PDAC. Budesonide, a first-line asthma therapy, reduced mesenchymal invasion in two-dimensional cultures at high micromolar concentrations through glucocorticoid receptor (GR)-independent mechanisms, while inhibiting proliferation in three-dimensional models and xenografts at low nanomolar concentrations through GR-dependent mechanisms. These effects were associated with metabolic reprogramming from glycolysis toward oxidative phosphorylation and upregulation of CDKN1C/p57Kip2. This microenvironment-dependent antitumor activity may help explain epidemiological observations of lower PDAC incidence among asthmatic patients and supports further investigation of budesonide as a candidate strategy for targeting stromal resistance [60]. Statins, including simvastatin and atorvastatin, have been evaluated in large observational cohorts and have been associated with reduced recurrence risk after resection (HR 0.75), potentially through inhibition of HMG-CoA reductase signaling and downstream modulation of CAF-associated RhoA/ROCK-mediated matrix stiffening [61,62]. Itraconazole has shown anticancer activity, with antiproliferative effect through Hedgehog pathway inhibition and reduction in α-SMA-positive myofibroblasts and hyaluronan-associated stromal barriers [63] as a potential therapeutic strategy for PDAC, including 5-fluorouracil-resistant pancreatic cancer cells. DSF/Cu showed significant cytotoxic activity in both parental and resistant BxPC-3 and CFPAC-1 cells, associated with modulation of ROS balance, reduced NRF-2 expression, altered HO-1 regulation, and changes in Akt/MAPK survival signaling. In a xenograft mouse model, oral DSF/Cu reduced tumor growth and NRF-2 expression. Overall, the study supports DSF/Cu as a promising preclinical strategy to overcome chemoresistance in PDAC, although clinical validation is still required [64].

At the end, two ongoing biomarker-driven repurposing programs, including decitabine in the ORIENTATE trial [65,66] and valproic acid plus simvastatin in the VESPA trial [67], further underscore the growing interest in repositioning pharmacologic agents to reprogram the PDAC stroma and enhance therapeutic susceptibility. One such agent capable of reprogramming the PDAC stroma is PXS-5505, which targets the tumor stroma by inhibiting the lysyl oxidase enzyme family responsible for collagen cross-linking and tumor stiffening. By reducing tissue stiffness, PXS-5505 normalizes the tumor microenvironment and enhances the penetration of chemotherapeutic agents, such as gemcitabine, thereby promoting the destruction of cancer cells. Preclinical studies have also shown that PXS-5505 markedly reduces primary tumor progression and metastasis, including the spread of cancer to secondary organs such as the liver, by up to 45%. When combined with standard chemotherapy, it increased median survival by more than 35% in aggressive mouse models [40]. All studies described in this paragraph are reported in Table 2.

### 3.4. Biotechnological Strategies to Overcome Stromal and Immune Access Barriers

**Figure 3:**
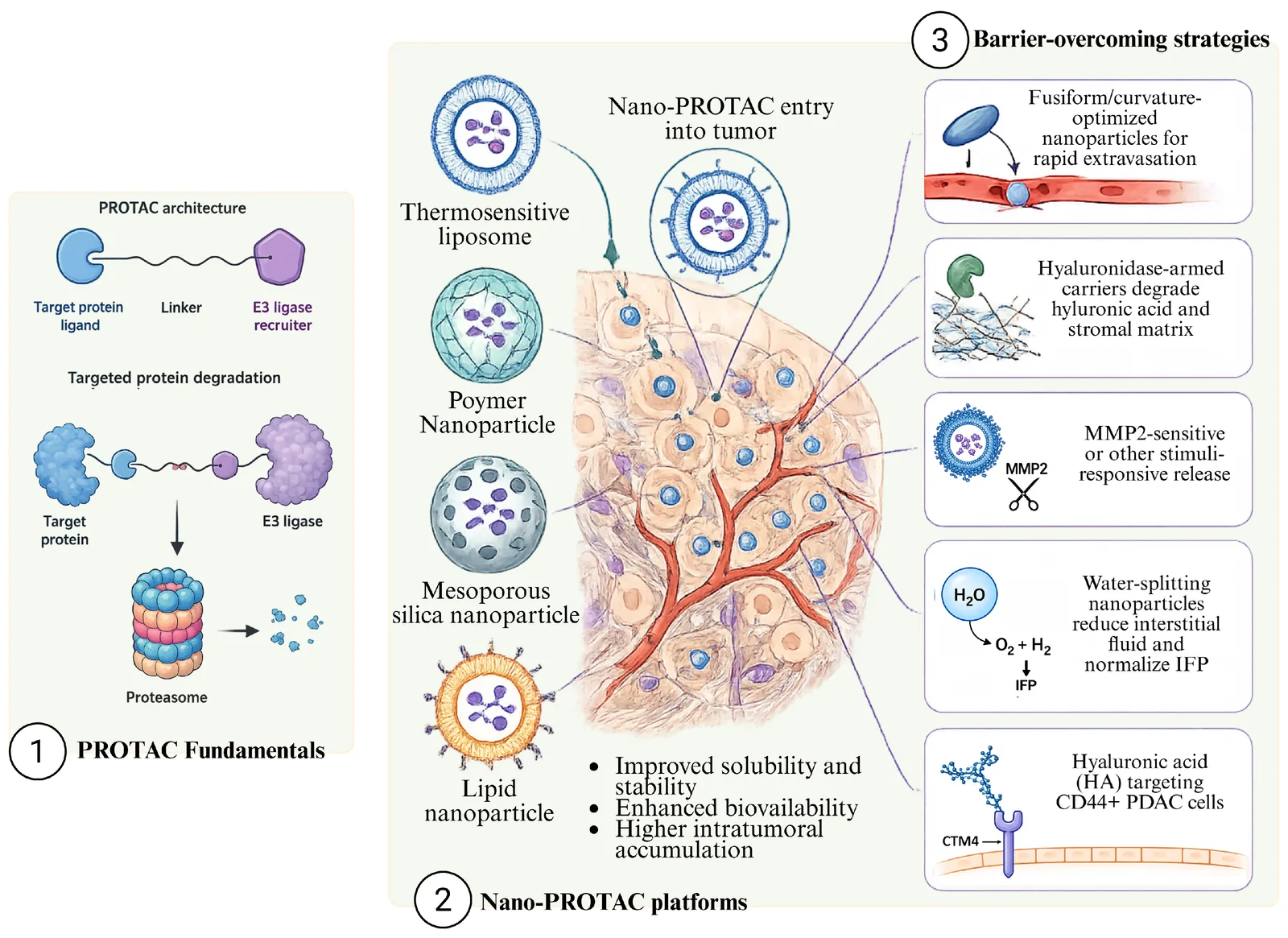
Nanotechnology-enabled PROTAC strategies to overcome stromal barriers in PDAC. Schematic overview of PROTAC-mediated targeted protein degradation and nano-PROTAC platforms designed to improve solubility, tumor accumulation, stromal penetration, and controlled release within the desmoplastic PDAC microenvironment.

Because stromal management alone may not fully overcome the pharmacological inaccessibility of PDAC lesions, biotechnology-based delivery systems are emerging as complementary strategies to enhance intratumoral drug access and molecular precision. The semaphorin axis illustrates PDAC stromal resistance, where microenvironmental cues organize protective niches that promote escape programs, including perineural invasion, macrophage polarization, CAF plasticity, immune exclusion, and inflammation [68]. These niches limit drug access via stiffness, vascular compression, pressure, hypoxia, and uneven distribution, supporting nanocarriers, ultrasound systems, and nano-PROTACs, though PROTACs remain constrained by size and pharmacokinetics in PDAC [69]. Nanotechnology acts not only as a formulation tool but also as a stromal navigation platform, improving PROTAC stability, circulation, tumor accumulation, controlled release, and penetration into hypoxic niches with high IFP. Consequently, nano-PROTACs extend microenvironment reprogramming to precise intratumoral delivery and targeted protein degradation [70]. However, their therapeutic translation remains limited by intrinsic physicochemical and pharmacokinetic constraints, including high molecular weight, poor water solubility, limited membrane permeability, suboptimal bioavailability, and dose-dependent loss of activity associated with the hook effect [71]. Nanotechnology enables PROTAC delivery via liposomes, polymeric, lipid, or mesoporous silica nanoparticles, improving solubility, stability, circulation, tumor accumulation, and controlled release. Supporting this, MR-HIFU with thermosensitive liposomal doxorubicin increased intratumoral accumulation and nuclear uptake ~23-fold in preclinical PDAC, informing clinical approaches such as PanDox [28,72]. Similarly, thermosensitive liposomal systems, including ThermoDox^®^-inspired formulations, enable ultrasound- or hyperthermia-triggered localized drug release, thereby improving intratumoral exposure. Post-2021 nano-PROTACs integrate targeted protein degradation with microenvironment-responsive delivery; for example, ARV-771-loaded glutathione-responsive nanoparticles enhance BRD4 degradation and induce ferroptosis [70,73]. In PDAC, fibrosis, vessel compression, and high interstitial pressure limit nanoparticle delivery despite improved pharmacokinetics. FAP-targeted small molecular drug conjugates (SMDCs) deliver cytotoxic agents to the tumor microenvironment via cleavable linkers, enabling stromal normalization while avoiding risks of indiscriminate fibroblast depletion [74]. Nanoengineering strategies aim to deliver PROTACs while reprogramming PDAC barriers, including curvature-optimized nanoparticles for enhanced extravasation, hyaluronidase-functionalized carriers, MMP2-sensitive POLY-PROTACs, and water-splitting nanoparticles that reduce interstitial pressure and improve intratumoral diffusion [73] (Figure 3).

Additional targeting strategies, such as hyaluronic acid-decorated carbon nano-onions directed toward CD44^+^ PDAC cells, have shown potential for delivering PROTACs to gemcitabine-resistant models, such as PANC-1, thereby amplifying degradation of oncogenic drivers, including BET proteins and AR [75]. Hybrid nanosystems that co-deliver PROTACs with antifibrotic or stromal-remodeling agents may further decompress tumor vasculature, reduce IFP, and expand the therapeutic distribution volume within PDAC lesions [76]. Looking forward, the next generation of nano-PROTACs is expected to move beyond passive encapsulation towards programmable, microenvironment-adaptive degradation systems. Emerging 2024–2026 concepts include mRNA-encoded or RNA-scaffolded nano-PROTACs for spatiotemporal control of degrader assembly, non-canonical E3 ligase recruitment to expand the degradable proteome, and strategies aimed at challenging PDAC drivers, such as KRAS [77]. Carrier-free nano-PROTACs and split and mix prodrug architectures may enable PDAC-selective activation in hypoxic, enzyme-rich, and high-IFP niches, reducing systemic exposure while maximizing local degradation potency. Despite their mechanistic appeal, nano-PROTAC platforms in PDAC remain largely preclinical. Major barriers include pharmacokinetic complexity, tumor penetration across highly heterogeneous desmoplastic regions, reproducible manufacturing, off-target degradation, payload stability, immunogenicity, and demonstration of clinically meaningful intratumoral target degradation. Consequently, these platforms should currently be viewed as enabling technologies rather than clinically validated stromal reprogramming strategies. Clinical translation of these approaches is supported by experience from PanDox Phase I protocols, including NCT04852367 [28]. In the future, ultrasound-triggered, stroma remodeling, and precision-targeted nano-PROTAC platforms may converge to transform PDAC from a delivery–refractory malignancy into a more pharmacologically accessible disease.

#### Cellular and Gene-Based Strategies for Immune-Desert PDAC

**Figure 4:**
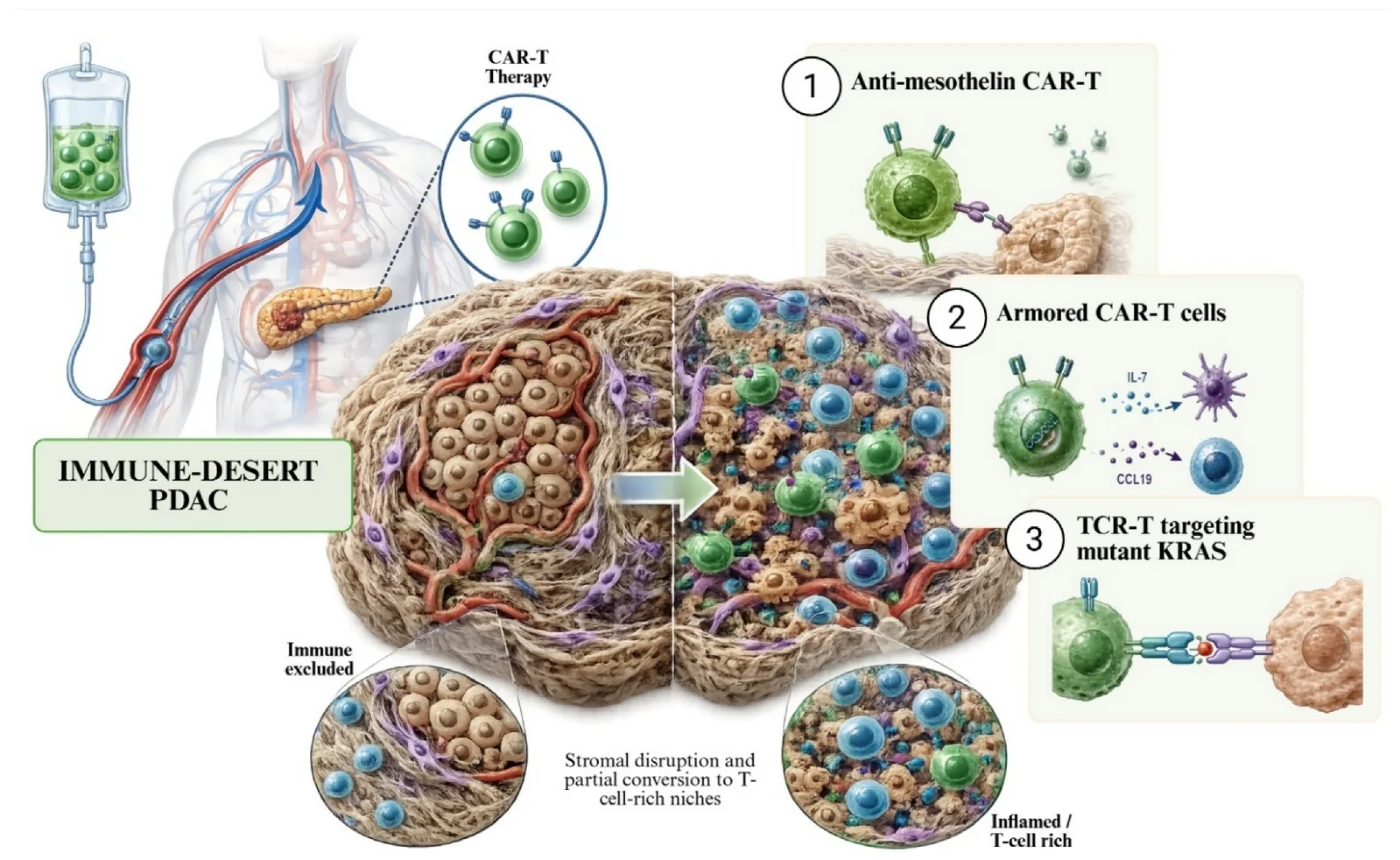
CAR-T and gene-based strategies to remodel immune-desert PDAC. Schematic representation of anti-mesothelin CAR-T cells, armored CAR-T platforms, and mutant KRAS-targeted TCR-T approaches designed to disrupt stromal barriers and promote conversion of immune-excluded PDAC into T cell-rich tumor niches.

Current clinical and gene-based trials employ CAR-T cells engineered to actively remodel the hostile tumor microenvironment, combining cellular therapies with stromal-targeting strategies and genetic checkpoint modulation to drive antitumor responses [78]. In a phase 1/2 trial, monthly infusions were safely administered to patients with advanced or resectable PDAC, with minimal treatment-related toxicity and evidence of long-term T cell persistence and antigen spreading. Disease control was higher in chemotherapy-responsive patients, and durable disease-free survival was observed in a subset of resected cases, supporting further evaluation of this multiantigen-targeted T cell therapy alone or in combination with other immunotherapies [79]. A phase 1 trial assessing the safety and feasibility of intravenous and local administration of anti-mesothelin CAR T cells in advanced PDAC demonstrated that CAR-T cells can traffic into pancreatic tumors and induce objective metabolic responses, although efficacy remains limited by rapid T cell exhaustion and stromal barriers [80]. At the same time, gene-modified CAR-T cells engineered to co-express stroma-remodeling modules (for example, FAP-targeted T cell engagers, or dual-targeting constructs against mesothelin and CD19) have shown enhanced elimination of PDAC tumor bulk and cancer-associated fibroblasts in preclinical models, highlighting that genetic rewiring of CAR-T cells can promote stromal disruption and partial conversion of immune deserts into infiltrated, T cell-rich niches [81]. Next-generation “armored” CAR-T cells enhance persistence and recruit endogenous immunity via cytokines (e.g., IL-7, CCL19). Checkpoint-edited or cytokine switch designs (PD-1–disrupted, TGF-β–resistant) preserve function, supporting CAR-T cells as local immune-remodeling platforms in PDAC [82]. Delivery route is another critical variable. Locoregional administration, including intratumoral, intraperitoneal, or regional infusion approaches, may bypass defective vascular trafficking and increase CAR-T exposure at tumor or peritoneal metastatic sites while potentially limiting systemic toxicity. This strategy is particularly relevant for PDAC, where dense desmoplasia and abnormal vasculature restrict systemic lymphocyte entry [83,84]. Beyond CAR-based recognition of surface antigens, TCR-engineered T cells targeting intracellular neoantigens represent an important gene-based extension for PDAC. Because KRAS mutations are truncal drivers in most PDACs, TCR-T approaches directed against HLA-restricted mutant KRAS epitopes may overcome some limitations of surface-antigen heterogeneity. Early clinical reports and ongoing trials of KRAS G12D- or G12V-specific TCR-engineered T cells indicate that driver-mutation targeting is feasible, although its applicability remains constrained by HLA restriction, neoantigen loss, and the same suppressive stromal barriers that limit CAR-T therapy [85,86]. Principal studies reported in this section are reported in Table 3. Future trials should integrate spatial and single-cell profiling to assess conversion of immune deserts into durable intratumoral immune responses (Figure 4).

### 3.5. Clinical Translation and Trial Landscape

The clinical translation of stromal normalization in PDAC is compelling but heterogeneous, spanning matrix remodeling, fibroblast reprogramming, vascular normalization, immune–stromal modulation, adenosine inhibition, and drug delivery. Trials report outcomes for PEGPH20, ATRA, losartan, CXCR4 inhibition, CD40 agonists, and CD73 inhibitors combined with chemotherapy [87]. In contrast, other approaches, including PanDox-like focused-ultrasound drug delivery platforms [28], spatially guided stromal biomarkers [88], nano-PROTACs [89], and some cellular immunotherapy strategies, remain mainly supported by translational, protocol-level, or preclinical evidence. The mixed results of completed trials highlight why biological rationale alone is insufficient in PDAC. PEGPH20, a hyaluronidase targeting hyaluronan to improve perfusion, failed to improve OS in HALO-301 despite earlier biomarker-enriched signals, underscoring limits of stromal depletion without precise selection and adaptive monitoring [90]. Conversely, ATRA-based stromal modulation was clinically feasible in the phase Ib STARPAC trial, where ATRA plus gemcitabine/nab-paclitaxel was tested as a pancreatic stellate cell-targeting strategy in advanced unresectable PDAC [53]. Losartan combined with FOLFIRINOX and chemoradiotherapy improved downstaging and R0 resection in unresectable PDAC, with translational data linking efficacy to modulation of pro-invasive, immunosuppressive programs, and microenvironmental remodeling [51]. Immune–stromal strategies show encouraging but non-definitive signals. In COMBAT, the CXCR4 antagonist BL-8040/motixafortide with pembrolizumab and chemotherapy demonstrated activity in metastatic PDAC, supporting CXCL12/CXCR4 targeting. CD40 agonists engage macrophage and antigen-presenting cell biology and are being tested with chemotherapy and checkpoint blockade [91]. In the randomized PRINCE trial, sotigalimab and/or nivolumab were evaluated with gemcitabine/nab-paclitaxel in first-line metastatic PDAC, providing an important benchmark for immune–stromal combinations [92]. More recently, the OPTIMIZE-1 phase 1b/2 study reported manageable safety and encouraging activity for the CD40 agonist mitazalimab combined with mFOLFIRINOX in untreated metastatic PDAC, supporting further confirmation in randomized settings [93]. Adenosine pathway targeting is also entering late-stage clinical translation [50,93]. In ARC-8, quemliclustat, a CD73 inhibitor, combined with gemcitabine/nab-paclitaxel with or without zimberelimab showed clinical and translational evidence of adenosine pathway modulation, including reduced adenosine-regulated NR4A expression and T cell activation in the tumor microenvironment [32]. The ongoing phase III PRISM-1 trial is now testing quemliclustat plus gemcitabine/nab-paclitaxel versus placebo plus chemotherapy in treatment-naïve metastatic PDAC [94]. PDAC trials show inconsistent outcomes partly because many did not account for stromal heterogeneity. Stroma varies between and within lesions in tumor–stroma ratio, fibrosis, hyaluronan, CAF subtype composition, stiffness, perfusion, immune cell localization, and spatial organization. Biomarker studies link this heterogeneity to clinical outcomes: tumor–stroma ratio combined with PD-L1 associates with nodal metastasis, survival, and mutational features, while fibrosis correlates with survival and can be estimated radiologically. Spatial immune profiling reveals a marked inter- and intratumoral variability [37]. Unstratified trials dilute benefit: matrix decompression fails without relevant matrix barriers, and immune conversion falters when lesions lack antigen presentation, T cell recruitment, or reversible CAF/myeloid programs. FAP-targeted radioligands and SMDCs show high stromal specificity and safety in early PDAC studies; ultra-targeted FAP agents seek optimized payload delivery with reduced systemic toxicity [95]. Future stromal-targeted PDAC trials should, therefore, be designed as biomarker-driven, mechanism-linked studies rather than empiric add-on combinations. Longitudinal biopsies should be embedded whenever feasible to distinguish true stromal remodeling from nonspecific treatment effect [96]. Imaging endpoints, including radiomics, elastography, perfusion imaging, and drug delivery readouts, should be integrated with tissue endpoints such as collagen organization, CAF activation, immune cell redistribution, T cell infiltration into tumor nests, and pharmacodynamic markers of drug penetration. The PanDox protocol illustrates this shift by directly testing whether focused ultrasound-triggered thermosensitive liposomal doxorubicin can enhance drug delivery to non-resectable pancreatic tumors, using post-treatment tumor sampling to assess intratumoral drug uptake [28]. Similarly, STARPAC2 is evaluating ATRA plus gemcitabine/nab-paclitaxel in a randomized phase II framework for locally advanced PDAC, reflecting the movement from early safety studies toward controlled stromal reprogramming trials [30]. Overall, stromal targeting in PDAC requires precision trials with biomarker-driven enrolment, longitudinal profiling, spatial analyses, and functional endpoints to distinguish reprogramming from perturbation.

## 4. Discussion

The evidence synthesized in this narrative review, informed by a structured literature search, supports a central conceptual shift: PDAC stroma should be viewed as a dynamic and functionally active compartment that resists indiscriminate depletion and may be better addressed through selective normalization [97], immune tumor microenvironment remodeling, improved intratumoral delivery, and biomarker-guided patient selection [98].

This shift must be viewed through a hierarchy of evidence: at the clinical level, ATRA-based reprogramming, losartan regimens, CXCR4/CD40/CD73 targeting, metabolic interventions, and technology-assisted delivery collectively demonstrate that the PDAC microenvironment is pharmacologically modifiable [52,99]. At the translational level, tumor–stroma ratio, fibrosis extent, CAF activation states, extracellular matrix stiffness, perfusion, immune exclusion, adenosine signaling, and spatial immune organization may capture complementary dimensions of the PDAC ecosystem [100]. No single biomarker is likely to be sufficient. Future classifiers should integrate histology, spatial profiling, transcriptomics, proteomics, radiomics, and functional delivery metrics to identify actionable stromal states rather than merely quantify stromal abundance [101]. Metabolic modulation is a key, incompletely defined aspect of stromal reprogramming shaped by hypoxia, nutrients, autophagy, redox, lactate, CAF activity, macropinocytosis, and adenosine signaling [102,103]. Strategies such as autophagy inhibition, high-dose ascorbic acid, ketogenic or nutrient-directed interventions, metformin-related pathways, and oxidative stress modulation may, therefore, influence both tumor–cell vulnerability and microenvironmental organization [104,105].

At the preclinical/mechanistic level, several emerging strategies are innovative but require cautious interpretation. Semaphorin signaling, particularly SEMA3 family members, may influence perineural invasion, macrophage localization, CAF behavior, immune trafficking, and basal-like aggressiveness [21,24,106]. Semaphorins should be viewed as emerging spatial regulators that may refine the taxonomy of PDAC microenvironments and identify distinct neural or immune–stromal niches [24].

A similar caution applies to nano-PROTACs and other advanced delivery platforms. Nano-PROTACs may improve delivery, but formulation alone is insufficient in PDAC unless vascular compression, interstitial pressure, dense matrix, and heterogeneous drug distribution are also addressed [107,108].

In this context, immune–stromal interactions are particularly relevant: CAF-derived chemokines, TGF-β, CXCL12/CXCR4, myeloid recruitment, macrophage polarization, Tregs, hypoxia, and adenosine collectively shape an immune-restrictive microenvironment [109]. In PDAC, effective immunotherapy will likely depend less on checkpoint blockade alone than on biomarker-guided combinations restoring T cell access, remodeling myeloid/stromal compartments, and improving vascular/metabolic conditions [110].

The next generation of stromal-directed PDAC trials must differ from earlier studies. Baseline stromal phenotyping—fibrosis/tumor–stroma ratio, CAF state markers, matrix stiffness, vascular perfusion, immune infiltration/exclusion, and pathway signatures (CXCR4, TGF-β, CD73, CD40) [111,112] should guide enrollment or stratification. Trials have to incorporate longitudinal tissue, liquid biopsy, imaging, or spatial profiling endpoints to confirm genuine microenvironmental reprogramming. Delivery platforms should demonstrate increased intratumoral deposition; stromal-normalizing strategies, improved perfusion, or reduced stiffness; immune stromal therapies, effector cell entry, and myeloid redistribution; and metabolic interventions, measurable changes in redox state, hypoxia, nutrient utilization, autophagy, or immune metabolic suppression. The selection of studies in this review was guided by their biological, clinical, and translational relevance to PDAC stromal reprogramming. Because the evidence base was heterogeneous and included clinical, observational, mechanistic, preclinical, and emerging biotechnological studies, no formal risk of bias assessment or meta-analysis was performed. The review may, therefore, be subject to selection bias, and the findings should be interpreted as a critical thematic synthesis rather than as a systematic estimate of the totality of available evidence. Overall, PDAC stroma should be viewed as a heterogeneous, therapeutically actionable ecosystem.

## 5. Conclusions

In conclusion, the evidence reviewed here supports a transition toward selective, mechanism-based stromal modulation guided by measurable microenvironmental features rather than by stromal abundance alone. Future clinical development should, therefore, prioritize biomarker-selected combinations and pharmacodynamic endpoints capable of demonstrating true stromal, immune, and delivery-related reprogramming. However, most stromal biomarkers have not yet been prospectively validated as predictive tools, and further progress will depend on standardized composite biomarkers and biomarker-enriched prospective trials. This strategy may help define when stromal targeting is beneficial, in which patients it is most relevant, and how it can be integrated more rationally into PDAC treatment.

## Figures and Tables

**Table 1:** Candidate biomarker domains for biomarker-guided stromal stratification in PDAC. Representative stromal features and biomarkers potentially associated with prognosis, therapeutic vulnerability, and response to stromal-targeted interventions. Future patient stratification will likely require the integration of multiple biological domains into composite stromal classifiers.

Representative Biomarkers	Biological Significance	Clinical Association	References
TSR, fibrosis	Stromal abundance and architecture	Low TSR is associated with lymph node metastasis and shorter disease-free survival; fibrosis is associated with poor drug penetration and aggressive disease	[37]
CAF state signatures, ITGB5	Stromal activation and biomechanical remodeling	Distinct CAF subsets may promote or restrain tumor progression; ITGB5 expression is associated with increased stromal rigidity	[38]
ECM stiffness	Physical barriers and immune exclusion	Correlates with CAF activation and worse outcomes	[40]
T cell infiltration/exclusion, spatial immune profiling, CXCL12/CXCR4, adenosine signaling	Immune accessibility of the TME	T cell exclusion is associated with poor prognosis; CXCL12–CXCR4 promotes CAF-mediated immune suppression; adenosine pathway activation is associated with T cell dysfunction	[42]
Intratumoral microbial signatures	CAF modulation and collagen deposition	Linked to fibrosis and immune exclusion	[39]

**Table 2:** Repurposed drugs targeting the stromal microenvironment in PDAC: clinical outcomes, biomarkers, and mechanistic insights.

Study/Drug	Phase	Clinical Trial Numbers	Key Outcomes	Biomarkers/Mechanistic Rationale	Reference
ATRA + gemcitabine/nab-paclitaxel (STARPAC)	Phase I	EudraCT: 2015-002662-23; NCT03307148.	PFS: 6.4 months; OS: 10.9 months. Post hoc median OS at RP2D: 11.7 months versus 8.5 months in the historical MPACT population; no HR or formal statistical comparison was reported	Stroma-rich tumors defined by TSR < 50%; CAF modulation; downregulation of RNA-processing proteins post-NAT	[54]
High-dose IV ascorbic acid + nab-paclitaxel + 5-FU/leucovorin + gemcitabine	Phase Ib	NCT03410030	Tolerable regimen; no MTD reached; PFS 7.1 months; OS 14.2 months versus historical 6–10 months	Glutathione depletion in KRAS-mutant cells; reduction in hepatic steatosis	[33]
Budesonide	Preclinical	/	Reduced invasion in 2D models at high micromolar concentrations; reduced proliferation in 3D models and xenografts at low nanomolar concentrations	GR-dependent and GR-independent effects; metabolic shift toward OXPHOS; CDKN1C/p57Kip2 upregulation	[60]
Medically supervised ketogenic diet + gemcitabine/nab-paclitaxel/cisplatin	Phase II	NCT04631445	Nutritional ketosis in 94%; median PFS 8.5 months; HR 0.53; median OS 13.7 months; HR 0.58; no incremental toxicity	β-hydroxybutyrate 0.5–3.0 mM; microbiome enrichment	[58]
Paricalcitol + pembrolizumab	Phase II	NCT03331562	Tumor microenvironment modulation with increased CD8-positive T cells and decreased Treg cells; no significant PFS benefit	Vitamin D receptor activation	[48]
Statins (for example simvastatin, atorvastatin)	Observational/meta-analytic evidence	/	Reduced recurrence risk after resection; HR 0.75	HMG-CoA reductase pathway; downstream RhoA/ROCK signaling; reduced CAF-associated matrix stiffening	[62]
Itraconazole	Preclinical/early clinical signal	/	Stable disease in 45% of advanced patients	Hedgehog pathway inhibition; reduction in α-SMA-positive myofibroblasts; hyaluronan-associated stromal barrier reduction	[63]
Hydroxychloroquine + gemcitabine + nab-paclitaxel	Phase II	NCT01506973	PFS 5.5 months	Autophagy inhibition; lysosomal drug sequestration blockade	[56]
Losartan + FOLFIRINOX	Phase II	NCT01821729 and NCT03563248	Reduced stromal stiffness and collagen deposition; improved drug penetration	High fibrosis; reduced collagen I; improved penetration	[51,52]
Disulfiram + copper	Preclinical	/	DSF/Cu induced cytotoxicity in parental PDAC cells and 5-fluorouracil-resistant counterparts; reduced tumor volume in xenograft mice; decreased NRF-2 expression in vivo; showed potential activity against chemoresistant PDAC models	NRF-2 downregulation; modulation of intracellular ROS levels; NRF-2-independent HO-1 expression; differential regulation of Akt and MAPK survival pathways; rationale based on oxidative stress modulation and disruption of chemoresistance-associated survival signaling	[64]
Decitabine (ORIENTATE)	Phase II	NCT05360264	Biomarker-driven strategy; response in refractory PDAC	KRAS-linked biomarker framework	[65,66]
Valproic acid + simvastatin (VESPA)	Phase II	NCT05821556	Increased PFS with gemcitabine/nab-paclitaxel and reduced toxicity	HDAC inhibition plus HMGCR inhibition; reduced CAF activation; EMT reversal	[67]
Metformin	Observational study	/	OS benefit in diabetic PDAC patients; HR 0.56	Diabetes status; AMPK activation; reduced mTOR signaling and stromal glucose uptake	[55]
PXS-5505	Preclinical PDAC/ Phase I in myelofibrosis	NCT04676529	Reduced desmoplasia and stiffness; improved perfusion; increased gemcitabine efficacy	Pan-LOX irreversible inhibition; reduced collagen cross-linking	[40]

**Table 3:** Principal cellular and gene-based clinical trial strategies for immune desert PDAC.

Study	Phase	Clinical Trial Number	Key Outcome	Reference
Activity of mesothelin-specific chimeric antigen receptor T cells against pancreatic carcinoma metastases	Phase I	NCT01355965	Efficacy: no objective responses; disease stabilization in 2/6 patients (PFS 3.8 and 5.4 months); in 1 patient, 69% reduction in total metabolic tumor volume with complete metabolic response of liver metastases at 1 month, but no effect on the primary pancreatic tumor	[78]
TACTOPS trial of autologous multiantigen-targeted T cells (PRAME, SSX2, MAGEA4, survivin, NY-ESO-1) in PDAC	Phase 1/2	NCT03192462	TACTOPS showed feasibility and favorable safety (1 treatment-related SAE/37 patients), with high disease control in chemotherapy-responsive advanced disease (84.6%) and durable disease-free survival in 2/9 resected patients; T cells persisted up to 12 months and correlated with benefit and antigen spreading	[79]
Anti-mesothelin CAR T cells (huCART-meso) in advanced pancreatic ductal adenocarcinoma (PDAC)	Phase I	NCT03323944	Post-infusion CAR T cells acquired exhaustion/dysfunction signatures (enrichment of GZMK^+^ phenotype and upregulation of ID3 and SOX4)	[80]
Study of mesothelin-targeting CAR T cells engineered to secrete a bispecific T cell-engaging molecule (TEAM) against FAP (on cancer-associated fibroblasts) and CD3 (on T cells)	/	Preclinical study	“MesoFAP CAR-TEAM” cells specifically bound both antigens, activated T cells, and eliminated PDAC tumor cells and stromal fibroblasts more effectively than CAR T cells targeting either antigen alone, including in models of primary and metastatic liver tumors	[81]
Single-patient case report of TCR gene therapy targeting the KRAS G12D neoantigen in metastatic pancreatic cancer	Exploratory/proof-of-concept study	NCT04146298	~72% regression of visceral metastases by RECIST v1.1, ongoing at 6 months, and engineered T cells remained detectable in peripheral blood	[85]

## Data Availability

No new data were created or analyzed in this study. Data sharing is not applicable to this article.
